# Protocol for culturing and imaging of ectodermal cells from *Xenopus*

**DOI:** 10.1016/j.xpro.2022.101455

**Published:** 2022-07-14

**Authors:** Nydia Tejeda-Muñoz, Julia Monka, Edward M. De Robertis

**Affiliations:** 1 Department of Biological Chemistry, David Geffen School of Medicine, University of California, Los Angeles, Los Angeles, CA 90095-1662, USA

**Keywords:** Cell Biology, Cell culture, Developmental biology, Microscopy, Model Organisms, Signal Transduction

## Abstract

The *Xenopus* embryo provides an advantageous model system where genes can be readily transplanted as DNA or mRNA or depleted with antisense techniques. Here, we present a protocol to culture and image the cell biological properties of explanted *Xenopus* cap cells in tissue culture. We illustrate how this protocol can be applied to visualize lysosomes, macropinocytosis, focal adhesions, Wnt signaling, and cell migration.

For complete details on the use and execution of this protocol, please refer to Tejeda-Muñoz et al. (2022).

## Highlights

• Quick, inexpensive protocol for culturing frog embryo animal cap cells
• Simple assay to visualize cell biological properties of living embryonic cells
• Studying animal cap cells to reveal lysosomal function and cell adhesion
• Animal cap cells for Wnt signaling and cell motility studies

## Abstract (editor highlights)

Publisher’s note: Undertaking any experimental protocol requires adherence to local institutional guidelines for laboratory safety and ethics.

## Graphical abstract

**Figure:**
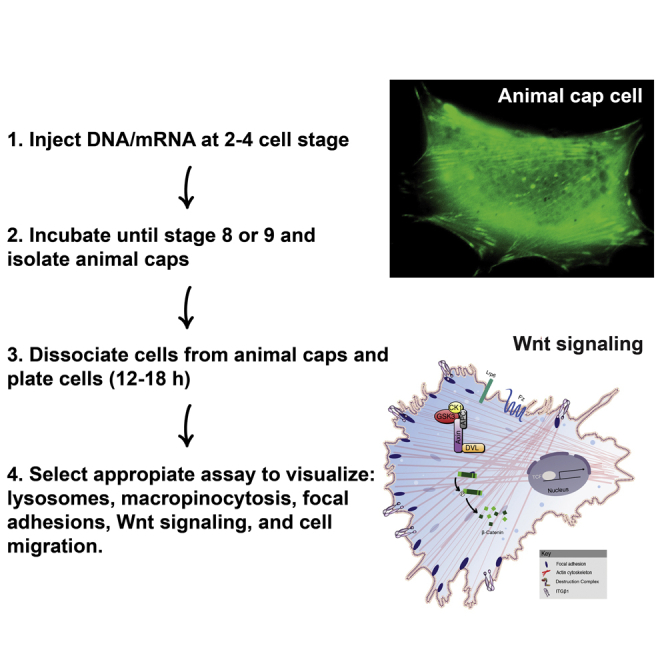

## Before you begin

*Xenopus laevis* animal cap cells are located in the animal (pigmented) pole of the blastula or early gastrula stage embryo (Ariizumi et al., 2009). By microinjecting the embryo during the early cleavage stages one can overexpress or deplete gene products with great ease, making these cells a favorite system for researchers (Asashima et al., 2009; Blum et al., 2015; Moriyama and De Robertis, 2018; Sosa et al., 2019). Only a few *Xenopus* permanent cell lines exist (Smith and Tata, 1991) and animal cap cells provide a system closer to the in vivo situation. The protocol below describes the specific steps for the visualization of living ectodermal cells. Cells cultured in L-15 medium (without serum) diluted to 50% with water survived for up to three days, provided they were attached to fibronectin-coated coverslips. Here we characterize the method and provide simple assays to visualize (1) lysosomes with lysosome-associated membrane protein 1 (LAMP1-RFP), (2) the multivesicular body marker CD63-RFP, (3) activated lysosomal Cathepsin D with the SiR-Lysosome reagent, (Marciniszyn et al., 1976), (4) macropinocytosis with added TMR-Dextran 70 kDa (Commisso et al., 2014; Albrecht et al., 2020; Tejeda-Muñoz et al., 2019; Redelman-Sidi et al., 2018), (5) focal adhesions labeled by microinjection with Tes-GFP DNA injection and, (6) F-actin during cell migration using LifeAct DNA expression (Tejeda-Muñoz et al., 2022). These assays have proven useful in the analysis of Wnt signaling (Tejeda-Muñoz et al., 2022). The protocol can in principle be adapted for any number of cell biological assays for which there are appropriate fluorescent protein tracers or gene depletion reagents.

### Institutional permissions

These experiments require vertebrate animals in order to prepare embryos; all experiments reported here have been approved by the UCLA Animal Research Committee in accordance to the Public Health Service Policy on Humane Care and Used of Laboratory Animals (ARC-1995-129).

### Preparation of explants

**Timing: 1 day**

**Figure 1:**
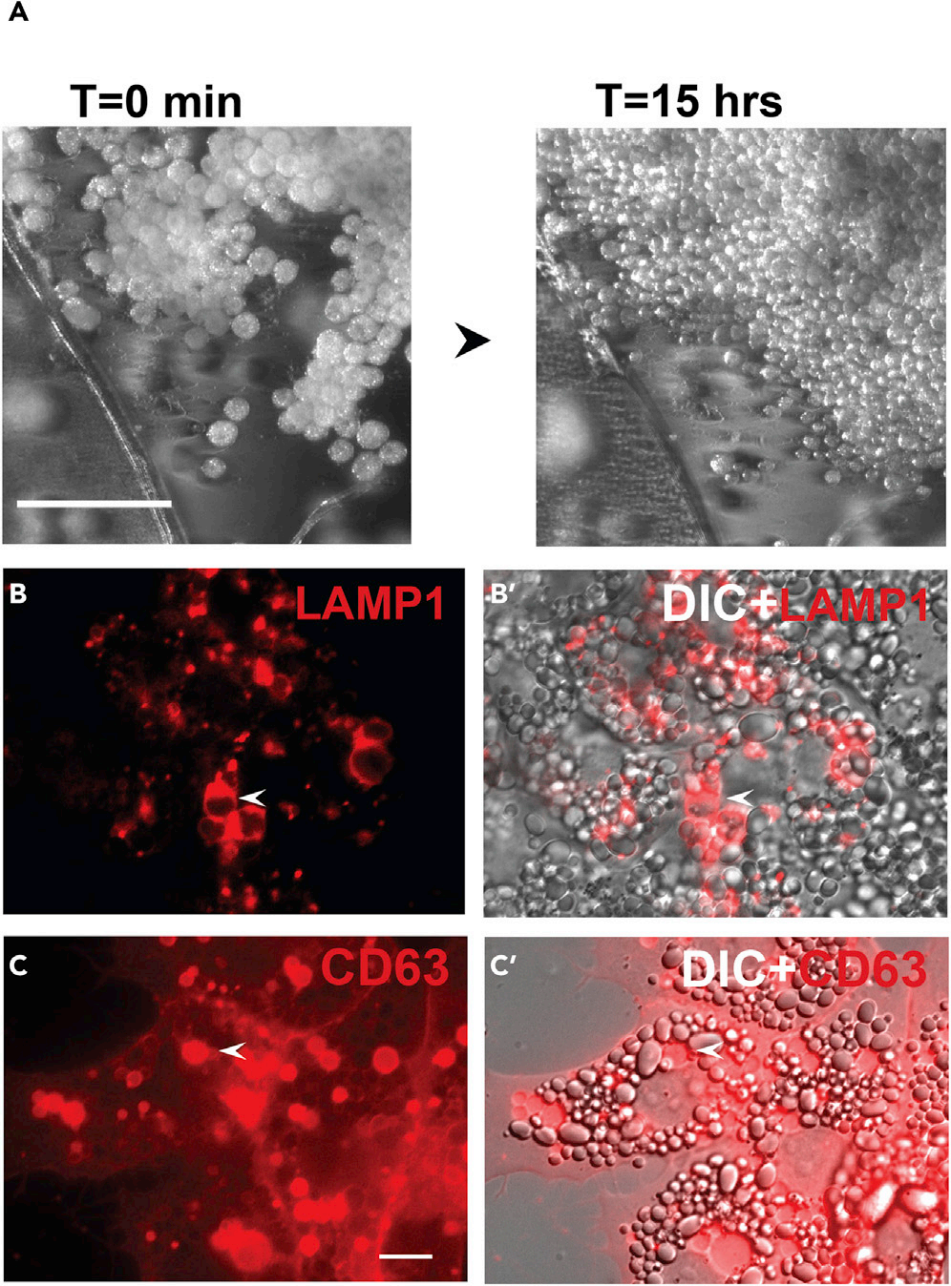
Dissociation of animal caps for lysosomal studies (A) Images from a movie of animal cap excised at blastula stage 8.5 plated in fibronectin and filmed for 15 h; note that cells divide in these conditions. Scale bar, 500 μm. (B–C′) Animal cap cells serve as a model for lysosomal studies using LAMP1-RFP or CD63-RFP as markers; the DIC visible light channel shows yolk platelets present in the embryonic cells. See also Methods video S1. Scale bar, 10 μm.

Preparation of animal cap single cells from *Xenopus* provides a very useful assay system (Sive et al., 2000). Embryos were prepared by in vitro fertilization with a *Xenopus laevis* testis suspension and cultured as previously described (Moriyama and De Robertis, 2018). Earlier work had used *Xenopus* gastrula mesodermal cells cultured in simple saline solutions and demonstrated a requirement of fibronectin or laminin for cell attachment (Nakatsuji, 1986; Smith et al., 1990). This section describes the preparation of ectodermal cells from *Xenopus* for use in culture using Leibovitz L-15 cell culture medium (Figure 1A). The cells can be treated with growth factors as well as drugs for studies of migration and cell-cell contact inhibition.

***Note:*** Animal caps can be dissected from manually dechorionated blastula-stage embryos with eyebrow hair knives; however, this is much slower than cutting the tissue with sharp Dumont #5 forceps.

1. Manually dechorionate the frog embryos at blastula stages 8 or 9 (staged according to Nieuwkoop and Faber, 1967) with forceps and dissect the animal cap region in 1 × MMR solution plastic Petri dishes covered with 2% agarose in H_2_O.

***Note:*** The animal cap cells normally develop into epidermis.

2. Transfer the animal cap explants into fresh 2% agarose (a thin coat of agarose in H_2_O) plates in 1 × MMR saline.
  a. Dissociate them manually by gently pipetting up and down 3–4 times. Avoid contact of cells with air. Use a pulled glass Pasteur pipette with a fire-blunted opening of about 1 mm.
3. Discard the more adherent, pigmented epithelial cells. Troubleshooting 1.

***Note:*** After the pipetting process, the outer pigmented epithelial cells remain attached to each other.

4. Plate the dissociated animal cap cells with a glass Pasteur pipette in L-15 medium (without serum) diluted to 50% with water in a 12-well culture dish containing circular fibronectin-coated coverslips for immunofluorescence.
5. For filming, plate the cells on fibronectin-coated glass-bottom chambers. Place cells from approximately 6 to 8 animal caps into each well. Troubleshooting 2.
  a. Sterilize the coverslips before adding them to the wells by autoclaving or immersing them in 70% ethanol under a sterile tissue culture hood. Allow the ethanol to evaporate and then place coverslips into individual wells of a 12-well culture plate.
  b. Add fibronectin to the same wells containing the coverslips used for cell adhesion and spreading (Nakatsuji, 1986). Use 10 μg/mL fibronectin for 30 min at 37°C (Sigma F4759).
  c. Wash coverslips with phosphate buffered saline (PBS, Gibco) 3 times and then add a volume of 1 mL of 1:1 L-15:H_2_O medium to each 12-well and add cells.
6. Incubate the cap cells at 15°C–20°C for 12–18 h. Individual ectodermal cells divide (Figure 1A) and remain attached while sibling embryos reach late neurula or tailbud stages.
7. Proceed with the respective protocol (movies or immunofluorescence) and treat the cells with chemicals or growth factors according to the chosen assay.

***Note:*** Animal cap dissociation can be accelerated using Ca^++^ Mg^++^-free MMR or a mixture of 1:10 MMR with or without divalent cations instead of mechanical disruption.

## Key resources table

**Table:** 

REAGENT or RESOURCE	SOURCE	IDENTIFIER
**Chemicals, peptides, and recombinant proteins**		

Digitonin	Sigma	Cat#300410
Sir-Lysosome	Cytoskeleton Inc	Cat#CYSC012
Tetramethylrhodamine Dextran (TMR-Dx) 70,000 kDa	Thermo Fisher Scientific	Cat# D1818
Lithium chloride (LiCl)	Sigma	Cat#L4408
10-cm dish	Thermo Fisher Scientific	Cat#174903
8-well glass-bottom chamber slides	ibidi	Cat#80827
Circular coverslips	ibidi	Cat#10815
Culture chambers #0 cover glass	E&G	Cat#GBD00003-200
Leibovitz L-15 cell culture medium	Thermo Fisher Scientific	Cat#11415064
Glutamine	Thermo Fisher Scientific	Cat#25030081
Bovine Serum Albumin (BSA)	Thermo Fisher Scientific	Cat#9048468
Pen-Strep antibiotics	Thermo Fisher Scientific	Cat#15140122
Triton X-100	Thermo Fisher Scientific	Cat#HFH10
Paraformaldehyde (PFA)	Sigma	Cat#P6148
Fibronectin	Sigma	Cat# F4759
PBS for cell culture applications	Gibco	Cat#10-010-023
PBS for immunostaining washes	Thermo Fisher Scientific	Cat#BP3994
Mounting Medium with DAPI	Abcam	Cat# ab104135
Protease Inhibitors	Roche	Cat#04693132001
Phosphatase inhibitors	Calbiochem	Cat#524629
mMESSAGE mMACHINE™ SP6 Transcription Kit	Thermo Fisher Scientific	Cat#AM1340

**Experimental models: Organisms/strains**		

*Xenopus laevis* (wild-type)	Xenopus I	N/A

**Recombinant DNA**		

LifeAct	IMSR	RRID: IMSR_EM:12427
pCS2-mGFP	Addgene	RRID: Addgene_14757
CD63-RFP	Addgene	RRID: Addgene_62964
xWnt8myc	Addgene	RRID: Addgene_16863

**Software and algorithms**		

ImageJ	NIH	http://imagej.nih.gov/ij/
Axiovision 4.8	ZEISS	http://Zeiss.com
Zen 2.3 imaging software	ZEISS	http://Zeiss.com

**Other**		

IM 300 microinjection pump	Narishige International USA, Inc	N/A
Axio Observer Z1 Inverted Microscope with Apotome	ZEISS	N/A

## Materials and equipment

### Preparation of macropinocytosis and lysosomal reagents prior to use

**Table:** 

Reagent	Traces	[Working]	Contents	No. of Exp. / Kit	λabs (nm)	λem (nm)	Probe
TMR-Dextran 70 kDa	Macropinocytosis	1 mg/mL	25 mg	25 coverslips	555	580	Intensiometric
SiR-Lysosome (1 mM)	Cathepsin D	1 μM	50 nmol probe 1 μmol verapamil	200 coverslips	652	674	Intensiometric

***Note:*** For all of the following reagents, limit light exposure throughout use.

SiR-Lysosome:

• For 1 mM stock concentration: dissolve a SiR-Lysosome vial in 50 μL anhydrous dimethyl sulfoxide (DMSO).
• Compound is unaffected by freeze-thawing. Do not aliquot as this will increase decay. Store SiR-Lysosome at −20°C until use, product is stable for at least three months.

TMR-dextran 70 kDa:

• For 1 mM stock concentration: Dissolve 25 mg TMR-dextran vial in 360 μL anhydrous DMSO.
• Compound is affected by repeated freeze-thaw cycles. For long-term storage, store aliquots of TMR-dextran 70 kDa at −20°C for several weeks.

1 × MMR solution (Marc’s Modified Ringers):

• 100 mM NaCl, 2 mM KCl, 2 mM CaCl2, 1 mM MgCl2, 10 mM HEPES pH 7.4.

### Microinjection and microscopy

Use M8 Wild dissecting microscopes for microinjection of Wnt pathway signaling components, multivesicular body (MVB) markers, focal adhesion markers, and mRNA or DNA encoding fluorescence markers in *Xenopus* embryos at the 2–4 cell stage. Microinjected DNA should not exceed 20 pg/cell. The Narishige microinjectors used were powered by an in-house air pressure line using Singer micromanipulators (Moriyama and De Robertis, 2018).

Live imaging was performed using a Zeiss Observer.Z.1 inverted microscope equipped with Apotome.2, an automated scanning stage, and Zeiss PEECON stage top incubation for Temperature/CO_2_ for cell culture. However, L-15 medium has the great advantage that it does not require CO_2_, and *Xenopus* cells develop at room temperature. Software image analyses can be achieved across an array of platforms including Imaris or the publicly available and free resource Image J.

## Step-by-step method details

This protocol is divided into three main sections that each offers a unique readout for culturing animal cap cells by monitoring lysosomal activity and macropinocytosis, cell migration and focal adhesions, as well as drug treatments that mimic Wnt signaling.

### Cultured animal cap cells for lysosomal studies

**Timing: 1 day**

**Figure 2:**
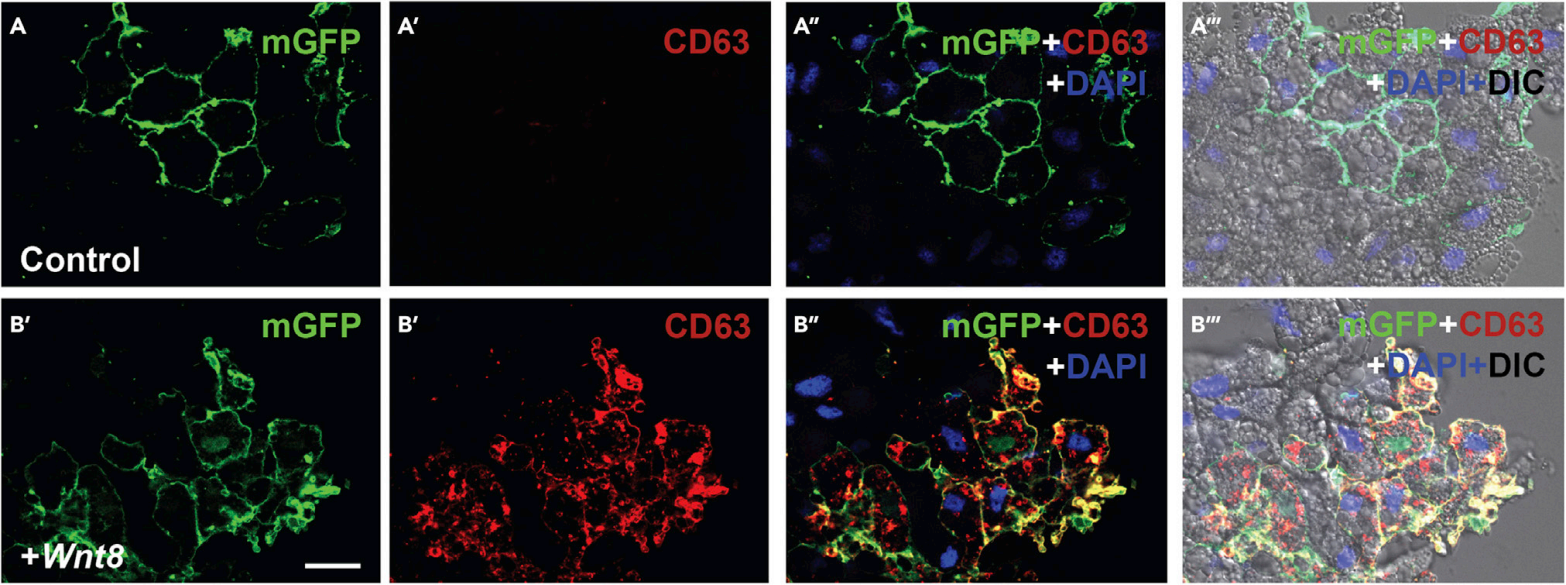
*Xenopus* animal cap cells showing that microinjection of 2 pg xWnt8 mRNA at the 2–4 cell stage greatly stabilizes CD63 lysosomes (A–A‴) Control animal cap injected with DNA encoding membrane GFP and the MVB marker CD63; uninjected cells serve as an internal control. (B–B‴). Co-injection of xWnt8 mRNA results in a striking stabilization of CD63, a marker of the intraluminal vesicles presents in multivesicular bodies and lysosomes. Canonical Wnt is a potent regulator of lysosome function. The images on the right panels show the merged red, green, blue and visible light channels. Scale bar, 10 μm.

**Figure 3:**
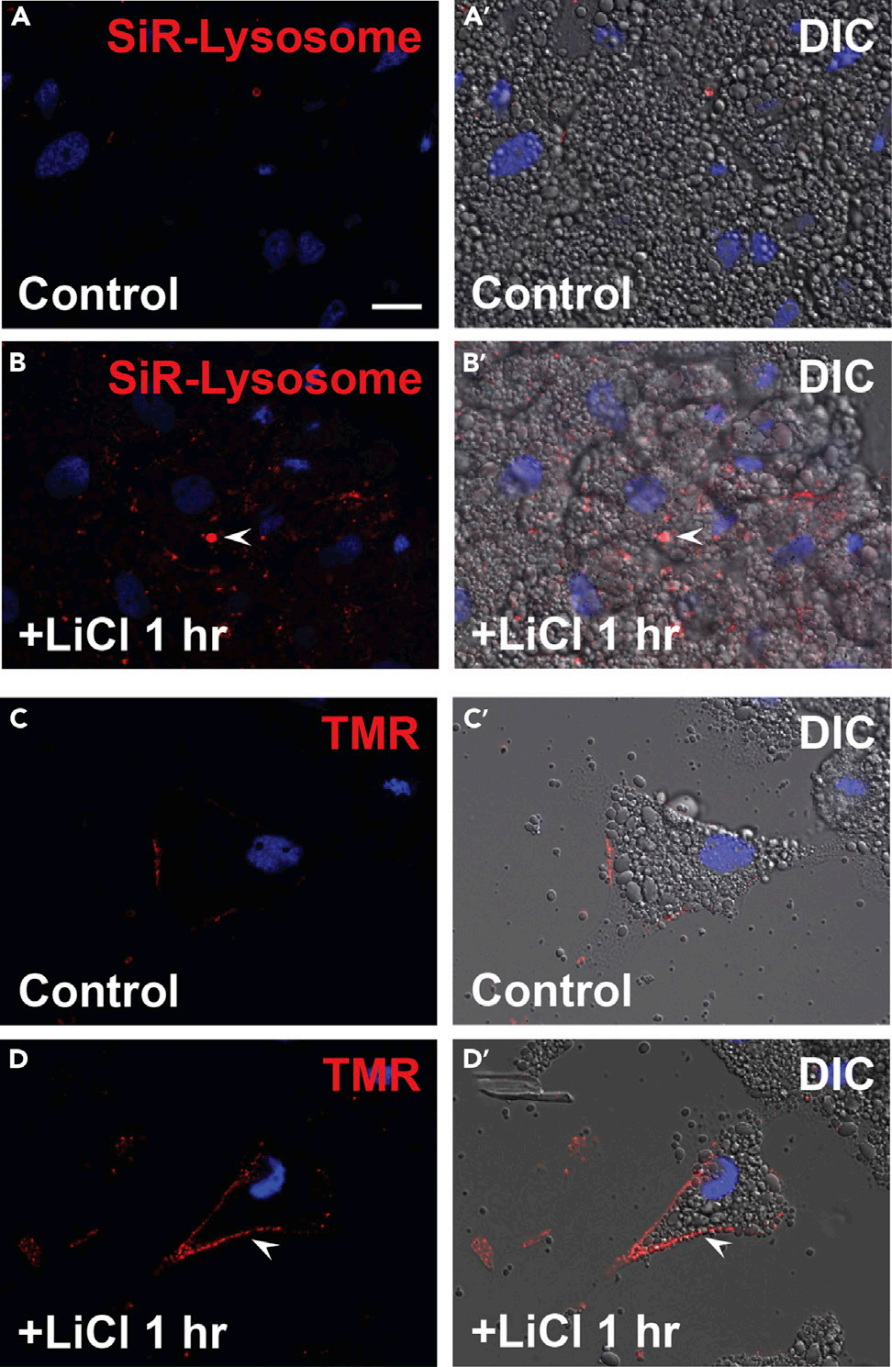
*Xenopus* animal cells display lysosomes and macropinocytosis activation after LiCl treatment, which mimics Wnt signaling by inhibiting GSK3 inhibition (A and A′) Control cells treated with SiR-Lysosome. (B and B′) LiCl treatment increases active lysosomes. Mimicking Wnt signaling with LiCl shows that lysosomes become more active. (C and C′) Untreated cells show low uptake of the macropinocytosis marker TMR-Dextran 70 kDa. (D and D′) LiCl treatment increases macropinocytosis (arrowhead). Scale bar, 10 μm.

This assay provides a powerful tool to study and visualize lysosome movements utilizing animal caps as an in vivo model in *Xenopus* using lysosome associated membrane protein 1 (LAMP-1-RFP) or the multivesicular body maker CD63-RFP after microinjection of DNA plasmids (Figures 1B–1C′). Animal cap cells also provide a good model to study how Wnt signaling stabilizes lysosome accumulation (Albrecht et al., 2021) using the CD-63-RFP multivesicular body marker co-injected with xWnt8 mRNA (Figure 2). SiR-Lysosome is a very useful reagent that specifically labels active lysosomes (Tejeda-Muñoz and De Robertis, 2022). It contains a fluorescent photostable silicon rhodamine (SiR) dye linked to a pepstatin A peptide (Marciniszyn et al., 1976). SiR-Lysosome binds to the activated and cleaved form of lysosomal Cathepsin D actin as a reporter of lysosomal activity. Its far-red emission (imaged in standard Cy5 filter sets) decreases phototoxicity and autofluorescence with compatibility for GFP- and mCherry-tagged proteins. By simply adding SiR-Lysosome to the culture medium, lysosomes containing active Cathepsin D can be visualized and their activation is increased by the Wnt mimic lithium chloride (LiCl) lysosomes (Albrecht et al., 2020; Tejeda-Muñoz and De Robertis, 2022). (Figures 3A–3B′).

Dilute SiR-Lysosome (1 mM stock) to a working concentration of 1 μM in culture medium (0.5 μL of the reagent per 500 μL L-15 medium 50% diluted with water).

***Optional:*** Addition of the calcium channel blocker, verapamil (working concentration of 1 μM), to SiR-Lysosome L-15 solution can increase signal intensity. This reagent is included with the commercial kit indicated in the Table above. Final concentrations of DMSO should not exceed 2% for treatment of cells, and a DMSO alone control should always be carried out.

For visualizing and studying lysosomes by microinjecting MVB markers in frog embryos by dissociating and culturing animal caps:

1. Fertilize *Xenopus laevis* embryos in vitro using excised testes and culture them in 0.1 × MMR solution. Then inject frog embryos with DNAs or mRNAs (in our examples 20 pg per blastomere of LAMP1-RFP or CD63-RFP DNAs) (Figures 1B–1C′), as well as CD63-RFP and membrane GFP DNAs ± Wnt8 mRNA) (Figure 2).

***Note:*** Perform the microinjection into the animal pole at 2–4 cell stage and culture them until midblastula stage 8.5 before excision (Nieuwkoop and Faber, 1967).

2. Dissect Animal caps at blastula stage 8.5–9 in 1 × MMR solution.
3. Dissociate the cap manually by pipetting the caps 3–5 times with a glass Pasteur pipette in 1 × MMR solution.
4. Plate the cells in L-15 medium diluted to 50% with H_2_0 (Tejeda-Muñoz et al., 2022) on fibronectin-coated coverslips in a 12-well plate (6–8 caps per condition).
5. Incubate the cells for 12–18 h between 15°C–20°C in normal atmosphere.
6. Wash with PBS 3 times to remove dead cells (Thermo Fisher Scientific).
7. Fix cells for 15 min using 4% Formaldehyde diluted in PBS (4% PFA) at 20°C with limited light exposure (0.5 mL per well).
8. Wash with PBS 3 times to remove PFA (0.5 mL per well).
9. Apply mounting medium with DAPI onto a glass microscope slide using 10 μL per coverslip. Avoid air bubbles in the pipette tip and place the coverslip carefully onto the mounting medium.
10. Microscopic examination of RFP and GFP and image acquisition was performed using a Carl Zeiss Axio Observer Z1 Inverted Microscope with Apotome and Nomarski differential interference contrast (DIC) and fluorescent filters for DAPI, FITC, and rhodamine. Images were acquired using 63× objectives and the Zeiss Zen software.
11. For image acquisition Fiji software is recommended. This can be downloaded at http://fiji.sc.

For live-cell analyses (as seen in Methods video S1): plate the dissociated animal cap cells (6–8 caps per condition) in a glass-bottom chamber (#0 cover glass, Cell E&G: GBD00003-200) for 12–18 h.

Methods video S1. Dynamic lysosomes in *Xenopus* animal cap cells, related to Cultured animal cap cells for lysosomal studies section, related to step 19 Movie showing that the lysosomal marker LAMP1-RFP can be visualized in frog embryo cells. Lysosomes are very dynamic and quite large. Scale bar, 10 μm.

After step 6 of the preparation of explants section described above:

12. Insert cell chamber for live imaging with conditions in the inverted microscope at 20°C (without CO_2_).
13. Image cells for 20 min or more with an image acquisition rate of 1 frame per 30 s. Images were collected with a Zeiss Observer.Z.1 inverted microscope with Apotome, DIC, and Colibri LED with green fluorescence filters using a 63× oil Plan-APOchromat objective. The microscope, including the fluorescence filters, was controlled by Axiovision 4.8 software. Both vertical and inverted microscopes were fully motorized.
14. Video editing was performed using the Adobe Premiere Pro CC 2021 software.
15. Cell viability was maintained for 72 h.

For SiR-Lysosome assay (Figures 3A–3B′), after step 6 of preparation of explants:

a. Incubate cells with SiR-Lysosome in 50% L-15 culture medium for 60 min at room temperature (500 μL per well). Troubleshooting 3.
b. Repeat steps 6–11 animal cap cell cultures as described in the cultured animal cap cells for lysosomal studies section.

For macropinocytosis with lysosomal tracers (Figures 3C–3D′), following step 6 of preparation of explants:

c. Dilute TMR-Dextran 70 kDa (1 mg/mL) in 50% L-15 culture medium and incubate cells for 60 min at room temperature, then continue with steps 6–11 in cultured animal cap cells for lysosomal studies section. Troubleshooting 4.

### Animal cap cells for migration and focal adhesions

**Timing: 1 day**

This section provides a way of monitoring changes in cell-cell adhesion, as well as cell migration, that can be triggered by activation of the Wnt pathway (Bachir et al., 2017; Nusse and Clevers, 2017) driving a cell growth program that can become inappropriately activated in cancer. Knowing how Wnt, focal adhesions, and cell migration cooperate will improve our understanding of embryonic development and tumorigenesis. In this improved protocol animal cap cells are able to adhere to fibronectin and display focal adhesions and actin stress fibers.

**Figure 4:**
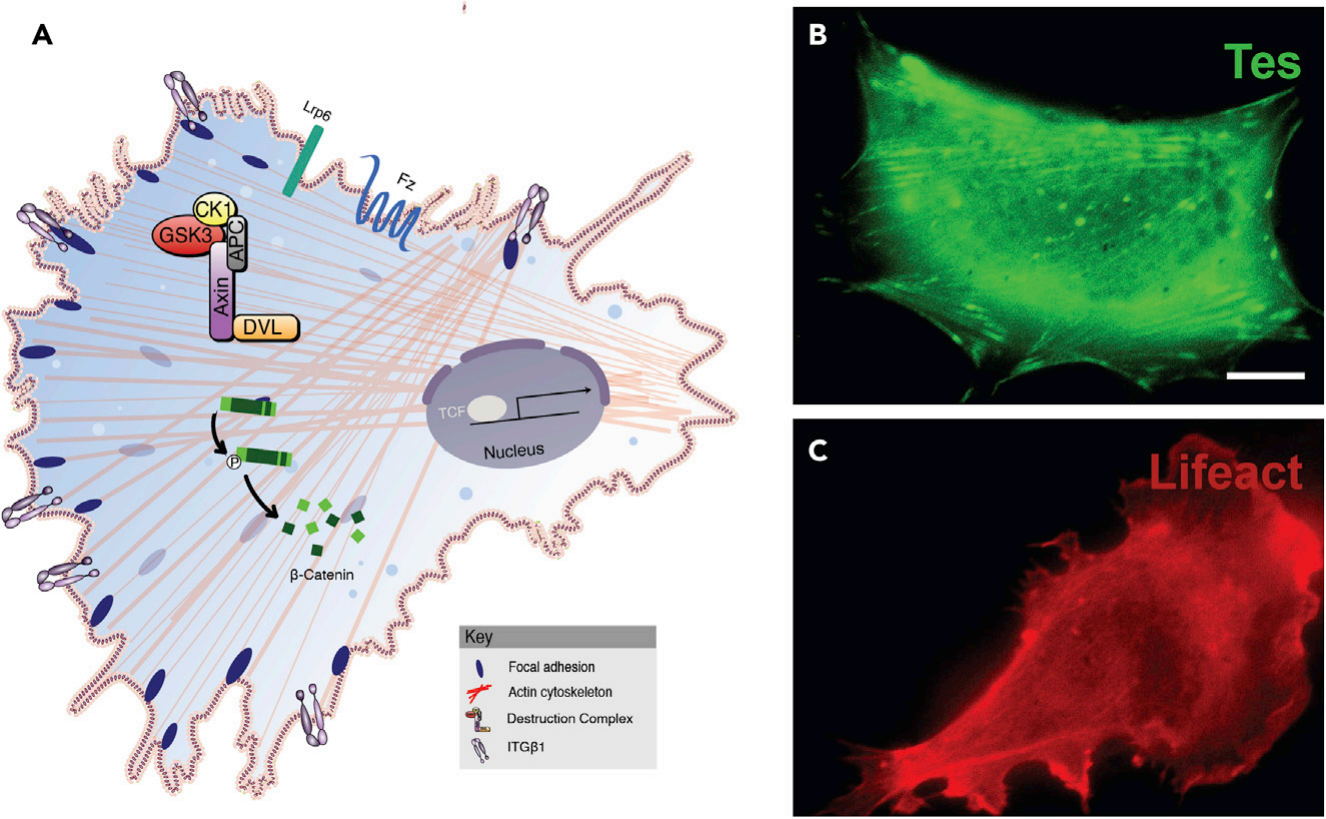
Focal adhesions in animal cap cells (A) Diagram showing actin cytoskeletal cables, membrane integrins, and focal adhesions in the leading edge of a migratory cell, as well as the β-catenin destruction complex that regulates canonical Wnt signaling. (B) *Xenopus* animal cap cells showing focal adhesions in the leading edge and actin cables marked by TES-GFP. Focal adhesions are critical for cell adhesion and migration. (C) A DNA plasmid encoding the F-actin tracer LifeAct (Gonagen) (in red) was microinjected into the animal pole at the 4 cell-stage (20 pg/injection). Scale bar, 10 μm. See also Methods video S2.

For live-cell analyses of focal adhesions:

Follow steps 1–6 in the preparation of explants section using frog embryos microinjected with 20 pg of DNA/cell at the 2–4 cell stage encoding the focal adhesion marker Tes-GFP or the F-actin tracer LifeAct (Figures 4A–4C). After 12–18 h following cell plating onto glass-bottom chambers:

16. Following any desired incubation with growth factor or drugs, wash 3 times with PBS.
17. Proceed with the movie generation by mounting a cell chamber for live imaging in the inverted microscope. Movie condition generation as previously described in the section about culturing animal cap cells for lysosomal studies.

### Drug treatment

**Timing: 1 day**

The activation of the canonical Wnt pathway is a driving force in many human cancers, especially colorectal, hepatocellular, and mammary carcinomas (MacDonald et al., 2009; Nusse and Clevers, 2017; Galluzzi et al., 2019; Tejeda-Muñoz et al., 2022, Tejeda-Muñoz and Robles-Flores, 2015). Wnt causes the stabilization and nuclear transport of newly-synthesized transcriptional regulator β-catenin, and the generally accepted view in the field is that the canonical effects of Wnt growth factors are caused by the transcription of β-catenin target genes. Recent studies indicate that Wnt is also a regulator of many other cellular physiological activities, such as macropinocytosis, endosome trafficking (Colozza et al., 2020), protein stability, lysosomal activity (Holland et al., 2020; Lawrence and Zoncu, 2019; Taelman et al., 2010; Tejeda-Muñoz et al., 2019), and cell adhesion (Tejeda-Muñoz et al., 2022).

For drug treatment assay of cultured animal cap cells:

After 12–18 h following cell plating onto glass-bottom chambers (steps 1–6 in the preparation of explants section):

18. Wash 3 times in 50% L-15 medium.
19. Proceed with the movie generation by mounting a cell chamber for live imaging in the inverted microscope at room temperature and imaging as described in the section on cultured animal cap cells for lysosomal studies under the live-cell analyses protocol.
20. Add drug treatments: GSK3 inhibitors (40 mM LiCl) or controls (40 mM NaCl or DMSO). Troubleshooting 5.
21. Start recording immediately.
22. Generate a movie in the same cells using the conditions used to film before drug treatment.

***Note:*** The use of antibodies for focal adhesions such as Tes, Zyxin, Vinculin can also be performed in cells plated on coverslips. In this case, following steps 1–6 in preparation of explants:

23. Wash 3 times with PBS (0.5 mL per well) to remove dead cells.
24. Fix cells for 15 min in 4% PFA diluted in PBS (0.5 mL per well).
25. Wash with PBS 3 times to remove PFA (0.5 mL per well).
26. Permeabilize cells with digitonin (6.5 μg/mL) for 10 min in PBS (0.5 mL per well).
27. Wash 3 times with PBS to remove detergent (0.5 mL per well).
28. Block cells for 1 h in blocking solution containing 5% bovine serum albumin in PBS to reduce background from nonspecific binding.
29. Incubate the samples with the primary antibody for 24 h at 4°C.
30. Wash 3 times with PBS (0.5 mL per well).
31. Incubate with secondary antibodies for 1 h at 20°C (0.5 mL per well) (ab150117 goat anti-mouse and ab150084 goat anti-rabbit are recommended).
32. Wash 3 times with PBS (0.5 mL per well).
33. Mount cells in mounting media with DAPI.
34. Dry slides in a dark area at 20°C for 16 h prior to imaging.

## Expected outcomes

The loss of normal cell polarity and adhesion caused by Wnt signaling activation is a fundamental step for tumor progression and metastasis. The crosstalk between Wnt signaling and focal adhesions is responsible for regulating adhesion, migration, and Wnt pathway component activities. Therefore, understanding how cell adhesion is affected in Wnt-driven cancers will be a useful tool in understanding the cellular dynamics during cell division, spreading, and adhesion, as well as in understanding how they can act as a tumor suppressor. For example Methods video S2, shows how mimicking Wnt activation using the GSK3 inhibitor LiCl treatment triggered increased cell motility. This illustrates that animal cap cells are a good model system to study cell movement, Wnt signaling, and cancer in embryo-derived cells.

Methods video S2. Animal cap as an in vivo model for Wnt signaling, related to Animal cap cells for migration and focal adhesions section, related to step 20 Mimicking Wnt signaling through GSK3 inhibition with LiCl strongly increased cell motility; note the actin-driven ruffling membrane motility at the leading edge of the cell in the direction of movement.

## Limitations

Problems with reagents such as SiR-lysosome or TMR-Dextran can occur as a result from incubation times that are too short, resulting in low fluorescence intensity. Possible contamination of the animal cap cells can also happen if sterile conditions are not kept, giving nonspecific artificial staining or high background. Staining between individual cells within a whole in vivo system during drug testing can be variable, requiring examination of multiple cells. The troubleshooting guidelines outlined below should be incorporated into the use of this protocol to help mitigate some of these risks. Environmental factors such as growth medium and drug treatment will require further optimization in independent lab settings. For this reason, the protocols included here describe methods with transparency and maximal details to increase the validity of data analyses.

## Troubleshooting

### Problem 1

Pigmented cells present in the animal cap.

### Potential solution

Avoid plating pigmented cells from the animal cap external layer as they quench fluorescence.

### Problem 2

Bacterial contamination of the animal cap cell cultures.

### Potential solution

Cleaning all the areas and materials with ethanol and using L-15 medium in sterile conditions to avoid cell contamination. Additionally, gentamicin (50 mg/mL) solution can be added to the culture medium.

### Problem 3

Low signal with lysosomal markers.

### Potential solution

Low fluorescence can result from high cell density on coverslips.

### Problem 4

Nonspecific artificial staining or high background in fixed cell imaging.

### Potential solution

Permeabilization with digitonin, or low concentrations of Triton X-100 0.15% (both diluted in PBS) can help reduce background fluorescence in immunostaining experiments. This procedure should be performed prior to blocking, with cells incubated with 0.5 mL of either 6.5 μg/mL digitonin or 0.15% Triton X-100 with 0.5 mL per coverslip in an individual well of a 12-well dish for 10 min. Following this, detergent should be removed with three washes of PBS (0.5 mL per well for each wash).

### Problem 5

Individual cells within a preparation system can show variability.

### Potential solution

Performing multiple independent experiments and quantitating the results in Fiji software.

## Resource availability

### Lead contact

Further information and requests for resources and reagents should be directed to and will be fulfilled by the lead contact, N Tejeda-Muñoz (ntejedamunoz@mednet.ucla.edu).

### Materials availability

This study did not generate new unique reagents.

## Data Availability

No data or code was generated in this study.

## References

[bib1] Albrecht L.V., Tejeda-Munoz N., Bui M.H., Cicchetto A.C., Di Biagio D., Colozza G., Schmid E., Piccolo S., Christofk H.R., De Robertis E.M. (2020). GSK3 inhibits macropinocytosis and lysosomal activity through the Wnt destruction complex machinery. Cell Rep..

[bib2] Albrecht L.V., Tejeda-Muñoz N., De Robertis E.M. (2021). Cell biology of canonical Wnt signaling. Annu. Rev. Cell Dev. Biol..

[bib3] Ariizumi T., Takahashi S., Chan T., Ito Y., Michiue T., Asashima M. (2009). Isolation and differentiation of Xenopus animal cap cells. Curr Prot Stem Cell Biol..

[bib4] Asashima M., Ito Y., Chan T., Michiue T., Nakanishi M., Suzuki K., Hitachi K., Okabayashi K., Kondow A., Ariizumi T. (2009). In vitro organogenesis from undifferentiated cells in Xenopus. Dev. Dynam..

[bib5] Bachir A.I., Horwitz A.R., Nelson W.J., Bianchini J.M. (2017). Actin-based adhesion modules mediate cell interactions with the extracellular matrix and neighboring cells. Cold Spring Harbor Perspect. Biol..

[bib6] Blum M., De Robertis E.M., Wallingford J.B., Niehrs C. (2015). Morpholinos: antisense and sensibility. Dev. Cell.

[bib7] Colozza G., Jami-Alahmadi Y., Dsouza A., Tejeda-Muñoz N., Albrecht L.V., Sosa E.A., Wohlschlegel J.A., De Robertis E.M. (2020). Wnt-inducible LRP6-APEX2 interacting proteins identify escrt machinery and trk-fused gene as components of the Wnt Signaling Pathway. Sci. Rep..

[bib8] Commisso C., Flinn R.J., Bar-Sagi D. (2014). Determining the macropinocytic index of cells through a quantitative image-based assay. Nat. Protoc..

[bib10] Holland L., Nielsen I., Maeda K., Jäättelä M. (2020). SnapShot: lysosomal functions. Cell.

[bib11] Galluzzi L., Spranger S., Fuchs E., Lopez-Soto A. (2019). WNT signaling in cancer immunosurveillance. Trends Cell Biol..

[bib12] Lawrence R.E., Zoncu R. (2019). The lysosome as a cellular centre for signalling, metabolism and quality control. Nat. Cell Biol..

[bib13] MacDonald B.T., Tamai K., He X. (2009). Wnt/β-catenin signaling: components, mechanisms, and diseases. Dev. Cell.

[bib9] Marciniszyn J., Hartsuck J.A., Tang J. (1976). Mode of inhibition of acid proteases by pepstatin. J. Biol. Chem..

[bib14] Moriyama Y., De Robertis E.M. (2018). Embryonic regeneration by relocalization of the Spemann organizer during twinning in Xenopus. Proc. Natl. Acad. Sci. U.S.A..

[bib15] Nakatsuji N. (1986). Presumptive mesoderm cells from xenopus laevis gastrulae attach to and migrate on substrata coated with fibronectin or laminin. J. Cell Sci..

[bib16] Nieuwkoop P.D., Faber J. (1967).

[bib17] Nusse R., Clevers H. (2017). Wnt/β-Catenin signaling, disease, and emerging therapeutic modalities. Cell.

[bib18] Redelman-Sidi G., Binyamin A., Gaeta I., Palm W., Thompson C., Romesser P., Lowe S., Bagul M., Doench J., Root D. (2018). The canonical Wnt pathway drives macropinocytosis in cancer. Cancer Res..

[bib19] Sive H.L., Grainger R., Harland R.M. (2000). Early Development of Xenopus laevis: A Laboratory Manual.

[bib20] Smith J.C., Symes K., Hynes R.O., DeSimone D. (1990). Mesoderm induction and the control of gastrulation in xenopus laevis: the roles of fibronectin and Integrins. Development.

[bib21] Smith J.C., Tata J.R. (1991). Chapter 32 Xenopus cell lines. Methods Cell Biol..

[bib22] Sosa E.A., Moriyama Y., Ding Y., Tejeda-Muñoz N., Colozza G., De Robertis E.M. (2019). Transcriptome analysis of regeneration during xenopus laevis experimental twinning. Int. J. Dev. Biol..

[bib23] Taelman V.F., Dobrowolski R., Plouhinec J.-L., Fuentealba L.C., Vorwald P.P., Gumper I., Sabatini D.D., De Robertis E.M. (2010). Wnt signaling requires sequestration of glycogen synthase kinase 3 inside multivesicular endosomes. Cell.

[bib24] Tejeda-Muñoz N., Robles-Flores M. (2015). Glycogen synthase kinase 3 in Wnt signaling pathway and cancer. IUBMB Life.

[bib25] Tejeda-Muñoz N., De Robertis E.M. (2022). Lysosomes are required for early dorsal signaling in the Xenopus embryo. Proc. Natl. Acad. Sci. U.S.A..

[bib26] Tejeda-Muñoz N., Albrecht L., Bui M., Robertis E. (2019). Wnt canonical pathway activates macropinocytosis and lysosomal degradation of extracellular proteins. Proc. Natl. Acad. Sci. U.S.A..

[bib28] Tejeda-Muñoz N., Mei K.-C., Sheladiya P., Monka J. (2022). Targeting Membrane Trafficking as a Strategy for Cancer Treatment. Vaccines.

[bib27] Tejeda-Muñoz N., Morselli M., Moriyama Y., Sheladiya P., Matteo P., De Robertis E.M. (2022). Canonical Wnt signaling induces focal adhesion and integrin beta-1 endocytosis. iScience.

